# Cytoreductive surgery (CRS) and hyperthermic intraperitoneal chemotherapy (HIPEC) for children and young adults: experience from two high volume centers

**DOI:** 10.1515/pp-2025-0011

**Published:** 2025-05-06

**Authors:** Maximilian Eckert, Michael Gerken, Jens M. Werner, Sebastian Blaj, Ferdinand Füsi, Niklas Bogovic, Hans J. Schlitt, Matthias Hornung, Pompiliu Piso, Miklos Acs

**Affiliations:** Faculty of Medicine, University of Regensburg, Regensburg, Germany; Tumor Center – Center for Quality Management and Health Services Research, University of Regensburg, Regensburg, Germany; Department of Surgery, University Medical Center Regensburg, Regensburg, Germany; Department of General and Visceral Surgery, Hospital Barmherzige Brüder, Regensburg, Germany; Department for Pediatric and Adolescent Surgery, Medical University of Graz, Graz, Austria

**Keywords:** cytoreductive surgery, hyperthermic intraperitoneal chemotherapy, peritoneal neoplasm, pediatric neoplasm, survival analysis, multimodal therapy

## Abstract

**Objectives:**

peritoneal surface malignancy in children is rare with a dismal prognosis. This bicentric study evaluated CRS with HIPEC in patients aged 2–25 years.

**Methods:**

Clinicopathological and treatment-related factors were retrospectively analyzed from 21 patients undergoing CRS and HIPEC between 2009 and 2022. Endpoints were feasibility, chemotherapeutic compound, complications, and overall survival (OS).

**Results:**

The mean age was 20.4 years. The mean peritoneal cancer index (PCI) was 12.8. Mean follow-up period was 6.8 years. Median overall survival time was 2.4. 5-year survival rate was 42.9 %. 76.2 % had primary and 23.8 % recurrent disease. The most common primary tumor locations were colon (33.3 %) and appendix (14.3 %). Adenocarcinoma was the most common histological subtype (71.4 %). Univariable Cox regression analysis showed significant impaired OS after previous chemotherapy (p=0.46) and incomplete cytoreduction CCR-2 (p=0.43). No perioperative mortalities occurred. The incidence of major complications was 24 %.

**Conclusions:**

Multimodal treatment can be considered in pediatric patients with peritoneal carcinomatosis. It presents a safe and feasible therapy with manageable complications and no perioperative mortality when performed by an experienced multidisciplinary team. Indication for CRS and HIPEC in children should be an individual decision by an interdisciplinary tumor board in the absence of better alternatives.

## Introduction

The peritoneum provides a favorable environment for the growth and spread of cancer cells, leading to the development of peritoneal carcinomatosis [1]. This process is closely linked to the peritoneal metastatic cascade, a sequence of events that facilitates the dissemination of tumor cells within the abdominal cavity [2]. The condition is often associated with a poor prognosis and limited treatment options, mainly because systemic chemotherapy is unable to sufficiently penetrate the peritoneal-blood barrier and to effectively target the peritoneal implants [3]. To improve survival of patients with peritoneal tumor seeding originating from intra-abdominal and pelvic malignancies CRS and HIPEC has progressively established its role as an effective treatment strategy in adults [4]. Although the median age for developing peritoneal carcinomatosis is 51 years in primary colorectal cancer (CRC) [5], 64 years for gastric peritoneal carcinomatosis [6], and 60 years for ovarian peritoneal carcinomatosis [7] the application of CRS and HIPEC in pediatric patients remains underexplored due to the rarity and clinical complexity of such conditions in this unique and fragile patient collective. Although growing evidence supporting the multimodal treatment strategy as viable therapeutic option in children with peritoneal surface malignancy [8] our knowledge is still scarce.

Designing and performing a prospective study in this subgroup of patients is exceedingly challenging due to the rarity of cases. However, with the increasing incidence of CRC in younger patients and the limited pediatric-specific data available, this study aims to provide valuable insights in the treatment of this vulnerable population. Furthermore poses a major objective whether, in addition to tumor biology characteristics, surgically modifiable factor in terms of macroscopically complete cytoreduction are associated with improved survival in these individuals.

For this purpose, the current study analyzes 13 years of experience with CRS and HIPEC in two major German surgical centers in Regensburg focusing on pediatric and young adult patients aged 2–25 years.

## Materials and methods

Data from two major centers from Regensburg comprising a total of 1,477 patients who underwent CRS and HIPEC for peritoneal surface malignancy between 2009 and 2022, were retrospectively analyzed. Among this extensive cohort, 21 patients aged 25 years or younger were identified and included in the study.

Written informed consent had been given by all included patients regarding data collection and use for research purposes prior to surgery. In case of minors, consent had been obtained from their respective legal guardians. The study was approved by the local Ethics Committee of University Regensburg (approval number: 23-3554-104).

### Therapy

Surgeries and peritonectomy procedures were performed in a standardized fashion according to Sugarbaker [9]. The completeness of cytoreduction (CCR) was scored as proposed by Sugarbaker [10]: CCR-0, CCR-1, CCR-2, and CCR-3 was defined as no residual disease, residual nodules measuring less than 2.5 mm, residual nodules measuring between 2.5 mm and 2.5 cm, and residual nodules larger than 2.5 cm, respectively [10]. The extent of peritoneal disease was assessed using the PCI [11]. Postoperative adverse events were categorized according to the Clavien–Dindo classification, and major complications were defined as≥Grade III [12].

Of the 21 patients included in this cohort, 14 (66.7 %) underwent preoperative chemotherapy, with 2 (9.5 %) of these cases representing recurrent disease. The number of cycles administered ranged from 3 to 21. A variety of chemotherapeutic regimens was utilized, with commonly applied protocols including FOLFOX (leucovorin calcium (folinic acid), fluorouracil, and oxaliplatin), XELOX (capecitabine (Xeloda) and oxaliplatin)/Avastin and VIDE (vincristine, ifosfamide, doxorubicin, and etoposide). For patients with recurrent disease, more targeted regimens such as FOLFOX with panitumumab and FOLFOXIRI (folinic acid, fluorouracil, oxaliplatin and irinotecan) with and without panitumumab were employed.

Post-operative chemotherapy was administered to 2 (9.5 %) of the 21 patients in the cohort following CRS and HIPEC, while 3 (14 %) additional patients received recommendations for chemotherapy from the tumor board. Common regimens included combinations of 5-FU, oxaliplatin, irinotecan, and epirubicin with cisplatin.

**Figure 1:**
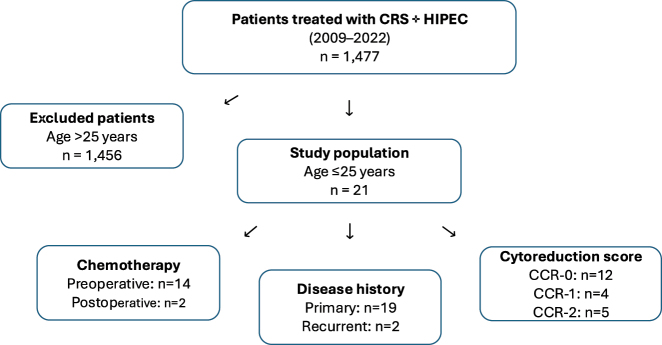
This flow chart summarizes the study design of inclusion criteria and treatment steps of the patients.

Two patients (9.5 %) in the cohort presented with recurrent disease. The first case involved a mucinous adenocarcinoma of the sigmoid colon with a PCI of 39 and a CC-2 score after both surgeries. The patient underwent two closed HIPEC procedures with oxaliplatin and 5-FU and received neoadjuvant chemotherapy before the second surgery (3 cycles of FOLFOX4 + panitumumab and 3 cycles of FOLFOXIRI + panitumumab). The second case involved adenocarcinoma of the ascending colon with a PCI of 8 at the initial surgery and 26 at recurrence. The patient underwent two closed HIPEC procedures with oxaliplatin, 5-FU, and leukovorin (first surgery) and received 6 cycles of preoperative chemotherapy FOLFOXIRI prior to the first surgery and adjuvant FOLFOXIRI afterward. Figure 1. summarizes the study design – as a flow chart-of inclusion criteria and treatment steps.

In the majority of cases the closed technique, utilizing unidirectional and bidirectional approaches, were employed with an exception, only one patient had undergone the open technique. HIPEC was performed immediately after cytoreductive surgery. The HIPEC procedure was performed either for 30 or 60 min duration with a goal intraabdominal temperature of 42 °C.

Given the extended duration of observation and the evolving nature of recommendations and emerging study findings during this timeframe, coupled with the multicenter nature of our study and different tumor entities, a diverse array of drug combinations were employed for the HIPEC procedure: cisplatin (75 mg/m2) and doxorubicin (15 mg/m2); oxaliplatin (300 mg/m2); oxaliplatin (300 mg/m2) and 5-flourouracil (400 mg/m2); oxaliplatin and doxorubicin (15 mg/m2); mitomycin C (15 mg/m2 and 30 mg/m2); 5-flourouracil (300 mg/m2) and calcium-folinate (40 mg/m2). The OS was defined as the period between the date of surgery, where CRS and HIPEC were performed, and the date of their death, measured in months.

### Statistical analysis

All statistical analyses were performed using SPSS Statistics (version 27, IBM, Armonk, NY). Continuous variables, such as age, body mass index (BMI), and PCI, were expressed as mean with range, and median with interquartile ranges (IQR), depending on the distribution. The normality of the data was assessed using the Shapiro–Wilk test. For normally distributed variables, comparisons between groups (e.g., male vs. female) were conducted using the Student’s *t*-test; non-normally distributed variables were compared using the Mann-Whitney-U test.

Categorical variables, including tumor histology and HIPEC procedures, were summarized as frequencies and percentages. Group comparisons for categorical variables were performed using Pearson’s chi-square or Fisher’s exact test where appropriate.

OS was estimated using the Kaplan–Meier method, and differences in survival between groups were assessed using the log-rank test. Univariable and multivariable survival analyses were performed using Cox`s proportional hazards model, adjusting for potential confounding variables such as age, sex, American Society of Anesthesiologists (ASA) score, histology, PCI, and previous chemotherapy. Hazard ratios (HR) with 95 % confidence intervals (CI) were reported. Statistical significance was set at a p-value of <0.05.

## Results

### Patient and tumor characteristics

This study involved 21 young patients, with a mean age of 20.4 years (range 2–25.7 years). There were 12 males (57.1 %) and 9 females (42.9 %) in the group. The BMI distribution revealed that 81 % of patients had a BMI below 25.0, while 14.3 % had a BMI between 25.0 and 29.9, and only 4.8 % had a BMI over 30.0. The BMI of the patients ranged between 15.5 and 31.5. The median BMI was 22.1 (IQR 19.5–23.5). The mean PCI was 12.8 (range 8–18) the most common histological subtype was adenocarcinoma, accounting for 71.4 % of cases. Among these, mucinous adenocarcinoma and standard adenocarcinoma were the most prevalent subtypes (each 23.8 %), followed by pseudomyxoma peritonei (14.3 %) and signet ring cell carcinoma (9.5 %). Other histologies included DSRCT (9.5 %), mesothelioma (4.8 %), yolk sac tumor (4.8 %), undifferentiated blastoma (4.8 %), and serous carcinoma (4.8 %). Primary tumor locations were varied, with the most common sites being the sigmoid colon (23.8 %) and appendix (14.3 %). Other primary sites included the stomach (9.5 %), ovary (9.5 %), and descending colon (9.5 %). Additional tumor origins included the pleura, and peritoneum accounting for 4.8 and 9.5 % respectively. The cohort also included one case of cancer of unknown primary (CUP). Among these, the sigmoid colon, appendix, and descending colon are classified as CRC sites, collectively representing 42.9 % of cases. These data are summarized in Table 1.

**Table 1:** Patient- and tumor characteristics.

		Count	Column N %
Age	<18	4	19.0 %
	18–20	6	28.6 %
	21–26	11	52.4 %
Sex	Male	12	57.1 %
	Female	9	42.9 %
BMI	<25.0	17	81.0 %
	25.0–29.9	3	14.3 %
	30.0+	1	4.8 %
ASA	I-II	14	66.7 %
	III-IV	7	33.3 %
Histology	Adenocarcinoma	5	23.8 %
	Mucinous adenocarcinoma	5	23.8 %
	Pseudomyxoma peritonei	3	14.3 %
	- LAMN	2	9.5 %
	- HAMN	1	4.8 %
	Signet ring cell carcinoma	2	9.5 %
	DSRCT	2	9.5 %
	Mesothelioma	1	4.8 %
	Undifferentiated blastoma	1	4.8 %
	Yolk sac tumor	1	4.8 %
	Serous carcinoma	1	4.8 %
Primary organ	Stomach	2	9.5 %
	Caecum	1	4.8 %
	Appendix	3	14.3 %
	Ascending colon	1	4.8 %
	Transverse colon	1	4.8 %
	Descendending colon	2	9.5 %
	Sigmoid colon	5	23.8 %
	Pleura	1	4.8 %
	Peritoneum	2	9.5 %
	Ovary	2	9.5 %
	CUP	1	4.8 %
Primary or recurrent	Primary	16	76.2 %
	Recurrent	5	23.8 %
Liver metastasis (resected)	No liver metastasis	16	76.2 %
	Single liver metastasis (resected)	5	23.8 %
PCI	0–9	9	42.9 %
	10–19	7	33.3 %
	20+	4	19.0 %
	Ns	1	4.8 %
	Total	21	100.0 %

ASA, American Society of Anesthesiologists; BMI, Body Mass Index; DSRCT, Desmoplastic Small Round Cell Tumors; PCI, Peritoneal Cancer Index; Ns, Non specified; LAMN, Low-Grade Appendiceal Mucinous Neoplasm; HAMN, High-Grade Appendiceal Mucinous Neoplasm.

### Treatment characteristics

Of the 21 patients, 61.9 % had undergone prior surgical interventions, and 66.7 % had received neoadjuvant chemotherapy before undergoing CRS and HIPEC. The procedures were primarily carried out using the closed unidirectional HIPEC technique in 76.2 % of cases, with closed bidirectional and open techniques accounting for 19.0 and 4.8 %, respectively. The average duration of surgery (excluding the HIPEC procedure) was 330.9 min, with a range from 83 to 565 min. A majority of patients (12, 57.1 %) achieved a complete cytoreduction (CCR-0), while 19.0 % had a CCR-1 score and 23.8 % had a CCR-2 score. Chemotherapeutic agents administered during HIPEC included oxaliplatin ± 5-FU in 47.6 % of cases and cisplatin ± doxorubicin in 33.3 %. Other agents used were mitomycin (9.5 %) and 5-FU ± folinat (9.5 %). The majority of patients (66.7 %) underwent a HIPEC procedure lasting 60 min, while the remaining 33.3 % underwent a 30-min HIPEC. Parietal peritonectomy was performed in 71.4 % of patients, with other organ resections (colon, small bowel, spleen, and liver) performed as needed. Further details on these treatment characteristics are provided in Table 2.

**Table 2:** Distribution of therapy.

		HIPEC	
		Yes	
		n	%
Previous surgery		13	61.9 %
Previous chemotherapy		14	66.7 %
CCR score	CCR-0	12	57.1 %
	CCR-1	4	19.0 %
	CCR-2	5	23.8 %
HIPEC procedure	Closed (unidirectional)	16	76.2 %
	Closed (bidirectional)	4	19.0 %
	Open	1	4.8 %
HIPEC duration group	HIPEC 30 min	7	33.3 %
	HIPEC 60 min	14	66.7 %
HIPEC substances	Oxaliplatin ± 5-FU	10	47.6 %
	Cisplatin ± doxorubicin	7	33.3 %
	Mitomycin	2	9.5 %
	5-FU ± Folinat	2	9.5 %
Parietal peritonectomy		15	71.4 %
Peritonectomy pelvis		13	61.9 %
Peritonectomy omental bursa		4	19.0 %
Peritonectomy right upper quadrant		11	52.4 %
Peritonectomy left upper quadrant		9	42.9 %
Anastomosis small bowel-small bowel		4	19.0 %
Anastomosis stomach-small bowel		2	9.5 %
Anastomosis small bowel-colon		5	23.8 %
Anastomosis colon-rectum		11	52.4 %
Anastomosis small bowel-rectum		2	9.5 %
Colostomy		1	5.6 %
Ileostomy		5	27.8 %
Permanent colostomy		1	5.6 %
Permanent ileostomy		1	5.6 %
Colon resection		13	61.9 %
Small bowel resection		7	33.3 %
Low anterior rectum resection		9	42.9 %
Splenectomy		7	33.3 %
Pancreas resection		2	9.5 %
Cholecystectomy		9	42.9 %
Greater omentectomy		13	61.9 %
Lesser omentectomy		7	33.3 %
Liver resection		7	33.3 %
Stomach resection		4	19.0 %
Hysterectomy		5	23.8 %
Resection other organs		7	33.3 %
	Total	21	100.0 %

CCR, Completeness of Cytoreduction; HIPEC, Hyperthermic Intraperitoneal Chemotherapy.

### Short-term outcomes

Postoperative complications were classified using the Clavien–Dindo system. A total of 23.8 % of patients had no complications (grade 0), while 47.6 % experienced grade II complications, including infections requiring antibiotics and other minor issues. Grade III complications, which required surgical, endoscopic, or radiological intervention, occurred in 23.8 % of patients. There were no grade IV complications. The perioperative mortality rate, defined as death from any cause within 30 days of surgery, was 0. The most common postoperative complications were pneumonia (14.3 %) and pleural effusion (9.5 %), while more severe complications like pneumothorax and peritonitis being rare (4.8 % each). The median hospital stay was 15 days, with a range of 10–28 days. ICU stays ranged from 1 to 7 days, with a median stay of 3 days. No cases of postoperative cholestasis were recorded, and only one patient (4.8 %) developed a urinary tract infection or anemia postoperatively. Reoperation was necessary in 16.7 % of the cases due to complications.

### Survival analysis

**Figure 2:**
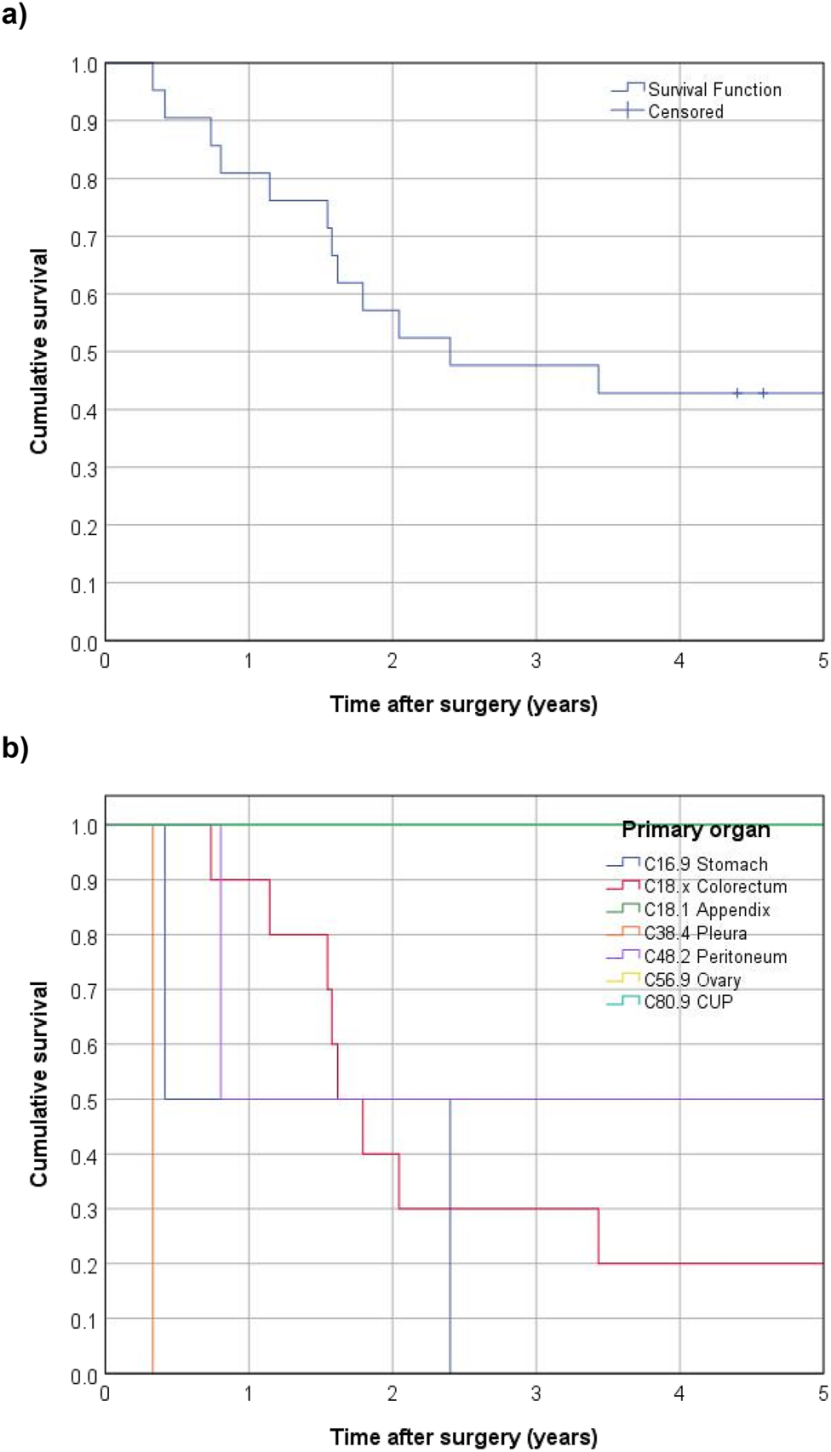
OS for the whole cohort (A) and according to the primary tumor origin (B) (Kaplan–Meier analysis).

**Figure 3:**
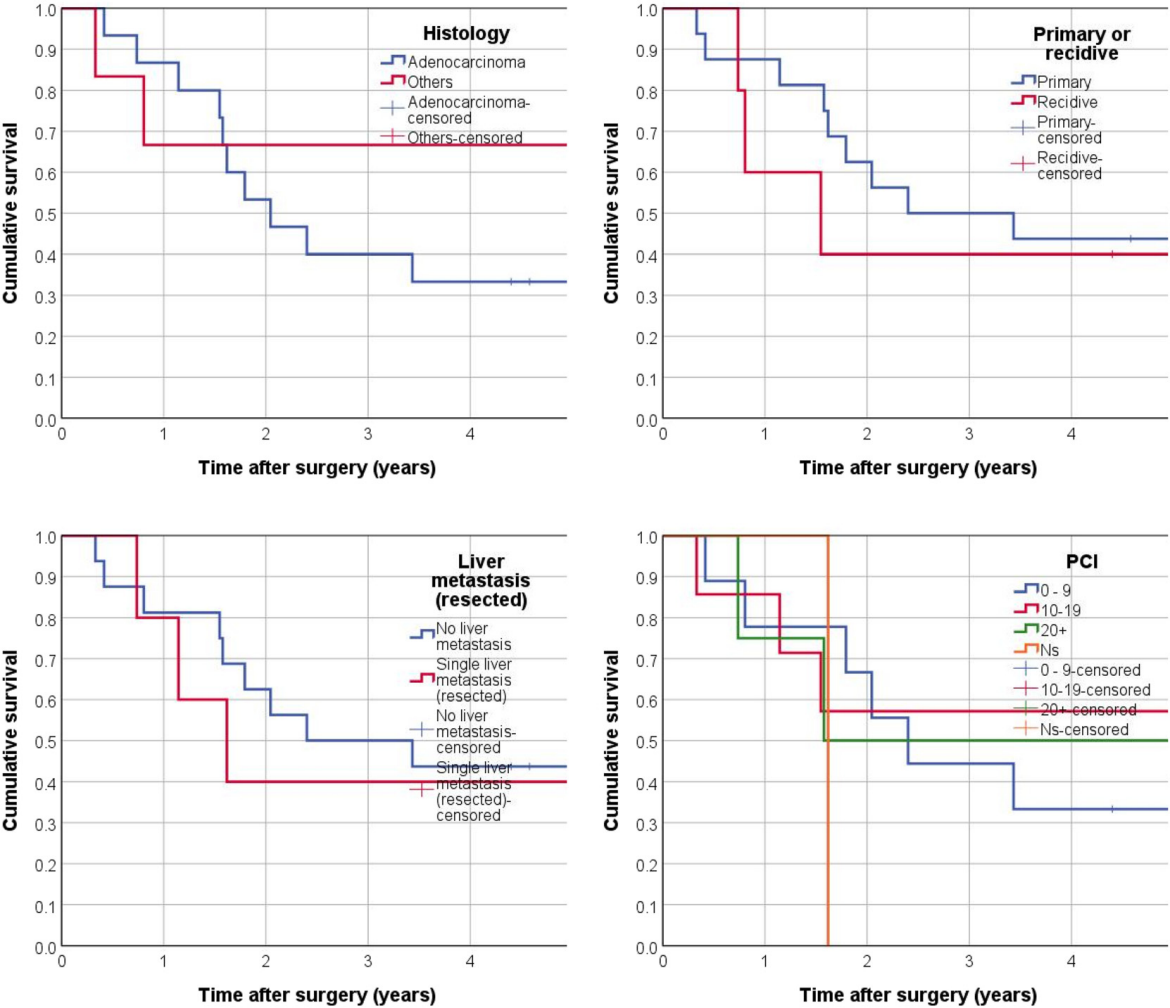
Overall survival according to tumor characteristics (Kaplan-Meier analyses). PCI, Peritoneal cancer index.

Out of 21 patients, 13 (61.9 %) died during the mean follow-up period of 6.8 years, resulting in a median overall survival time of 2.4 years (28.8 months) and a 5-year survival rate of 42.9 % (Figure 2). Out of the 21 patients 10 (47.6 %) had had peritoneal metastases delivered from colorectal origin with a median survival rate of 1.6 years. Survival data are shown in Figure 1 based on the tumor primaries. Kaplan-Meier analyses, and univariable and multivariable Cox regression analyses were performed to evaluate the impact of various prognostic factors on survival. The factors analyzed included age, sex, BMI, ASA score, histology, primary vs. recurrent disease, liver metastasis, and PCI. None of these factors were found to significantly affect survival outcomes (Figure 3). The survival data showed no significant difference between age groups (<18 years, 18–20 years, and 21–26 years) or between males and females. Similarly, BMI, ASA score, and tumor histology did not significantly impact survival.

**Figure 4:**
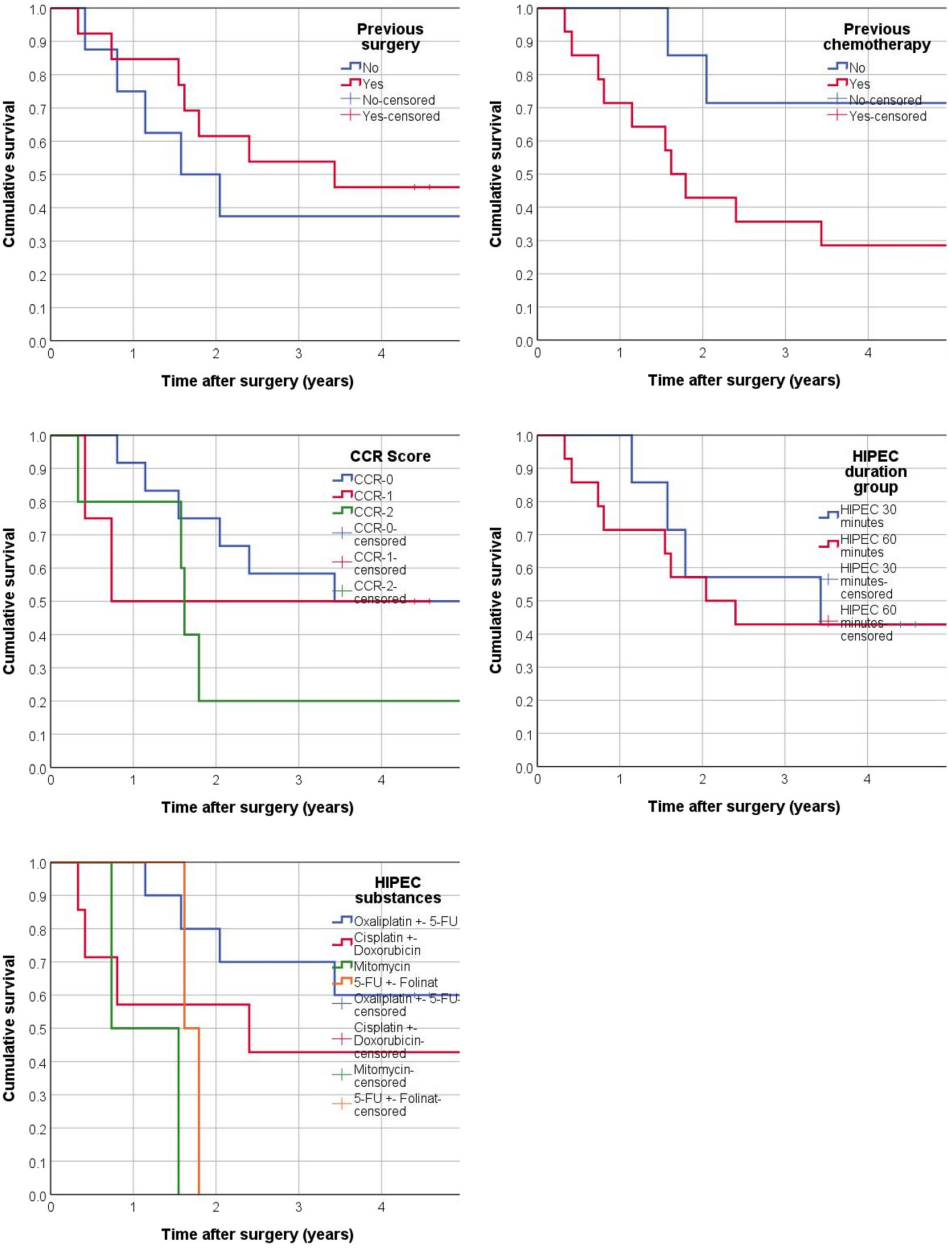
Kaplan–Meier analyses: none of the factors were significant at 0.05 level except previous chemotherapy (p=0.46) and CCR (p=0.43) with worse survival in patients with previous chemotherapy and CCR-2 vs. CCR-0. Two patients with mitomycin showed the lowest survival when comparing substances applied in HIPEC (p=0.003 compared to oxaliplatin-based HIPEC protocol).

In terms of treatment-related factors, previous surgeries, the specific HIPEC technique used (closed unidirectional, closed bidirectional, or open), and the type of chemotherapy agents administered during HIPEC were also analyzed in the uni- and multi-variable Cox regression and found to have no significant association with survival. However, patients who underwent previous chemotherapy showed worse survival compared to those without previous chemotherapy (p=0.046), and patients with a CCR-2 score had significantly shorter survival compared to those with a CCR-0 score (p=0.043) (Figure 4). No other treatment-related factors showed a significant impact on survival outcomes. These survival analyses are comprehensively presented in Table 3.

**Table 3:** Overall survival according to patient and tumor characteristics (Cox-regression). In uni- and multivariable Cox regression none of the prognostic factors proved to be significant. Including all therapy variables into the multivariable Cox proportional hazard model resulted in missing convergence of the coefficients. Including previous chemotherapy and CCR into the model, adjusting for sex, ASA, histology, liver metastasis and PCI, the effect of previous chemotherapy and CCR was still observed, but not significant.

Variable	Category	Univariable regression				Multivariable regression			
		HR^a^	Upper 95 %-CI	Lower 95 %-CI	p-Value	HR^a^	Uppe 95 %-CI	Lower 95 %-CI	p-Value
Age	<18	1.000				1.000			
	18–20	0.847	0.189	3.799	0.828	2.333	0.056	97.481	0.656
	21–26	0.774	0.192	3.119	0.719	1.651	0.040	67.442	0.791
Sex	Male	1.000				1.000			
	Female	1.116	0.374	3.336	0.844	5.000	0.445	56.167	0.192
BMI	<25.0	1.000				1.000			
	25.0–29.9	1.897	0.509	7.065	0.340	8.688	0.242	311.470	0.236
	30.0+	–	–	–	–	–	–	–	–
ASA	I-II	1.000				1.000			
	III-IV	0.695	0.213	2.268	0.547	0.231	0.030	1.806	0.163
Histology	Adenocarcinoma	1.000				1.000			
	Others	0.384	0.083	1.770	0.220	0.269	0.028	2.587	0.256
Primary or recurrence	Primary	1.000				1.000			
	Recurrence	1.290	0.352	4.727	0.701	0.971	0.140	6.712	0.976
Liver metastasis (resected)	No liver metastasis	1.000				1.000			
	Single liver metastasis	1.094	0.300	3.999	0.891	6.544	0.319	134.255	0.223
PCI	0–9	1.000				1.000			
	10–19	0.877	0.246	3.132	0.840	0.566	0.043	7.502	0.666
	20+	0.772	0.154	3.863	0.753	2.393	0.249	23.025	0.450
	Ns	2.120	0.243	18.534	0.497	0.406	0.004	38.132	0.697

^a^HR, Hazard ratio; CI, Confidence interval; ASA, American Society of Anesthesiologists; BMI, Body Mass Index; PCI, Peritoneal Cancer Index.

Seven patients (33 %) out of 21 were long-term survivors defined by ≥5 years survival from multimodal therapy. All the patients had had primary disease from various tumor origins. Except of one (4.8 %) of these patients underwent macroscopic complete tumor resection. The survival data according to tumor origin and characteristics of long term survivors are provided in Table 4.

**Table 4:** Characteristics of long term survivors.

Alter, Years	ASA	Histology	PCI	CCR-score	Prev CHT	Primary (P)/Recurrent (R)	Overall survival in years
24	3	Desmoplastic small round cell tumor of the peritoneum	22	CCR-0	Yes	P	5.74
23.8	2	Mucinous adenocarcinoma of the sigmoid colon	17	CCR-2	Yes	P	5.33
24.5	2	Pseudomyxoma peritonei (HAMN)	21	CCR-0	No	P	5.58
25.6	3	High-grade serous ovarian cancer	12	CCR-0	No	P	8.99
21	1	Yolk sac tumor	12	CCR-0	No	P	10.47
20.4	2	Mucinous adenocarcinoma of the sigmoid colon	8	CCR-0	Yes	P	8.13
2	2	Undifferentiated blastemal tumor	3	CCR-0	No	P	5.99

HAMN, High-Grade Appendiceal Mucinous Neoplasm; ASA, American Society of Anesthesiologists; CHT, Chemotherapy.

## Discussion

The experience in pediatric patients with peritoneal carcinomatosis using multimodal therapy is very limited worldwide, where a recent review by Byrwa et al. from 2023 included a total of 264 pediatric patients. Similarly, in our patient collective of 1,477, we were only able to retrospectively select 21 patients representing 1.4 % of the total collective.

The median OS of 2.4 years and a 5-year survival rate of 43 % in our cohort highlight the potential of CRS and HIPEC as a treatment option for patients with peritoneal carcinomatosis. Similar studies report OS rates in pediatric patients between 64 and 71 % for various tumor types [13], 14]. Nonetheless, due to the small number of cases, most studies report tumor-biologically different patients with different prognoses, like our report [8]. Attempting to homogenize the heterogenity, we further mapped the most common tumor histology CRC in the current study of 10 patients (48 %) with a median survival of 1.6 years (CI 95 % 1.283–1.953) and a median PCI of 12 which could serve as a benchmark for further studies. We also report seven patients (33 %) with long term survival (5–10 years) after multimodal treatment from which 2 patients with prognostically unfavorable colorectal histology.

While the incidence of CRC, which represents the dominant histology in our cohort, has declined overall due to the widespread use of screening colonoscopy and increased awareness for cancer prevention, a contrasting trend has been observed in younger patients [15]. Between 1990 and 2016, the incidence of CRC in young patients aged 20–29 years in Europe rose significantly from 0.8 to 2.3 cases per 100.000, with colon cancer increasing at an annual rate of up to 9.3 % in later years, while rectal cancer growing steadily at 3.5 % annually throughout the period [16]. Notably, prior research has identified young age as a potential adverse prognostic factor, attributed to more aggressive tumor biology, poor differentiation, and advanced-stage presentation at diagnosis [17].

Furthermore, prognostic factors significantly influencing survival in adult patients with peritoneal surface malignancy include the PCI and CCR score [18], 19]. Even though there is no uniform data on this in young patients, Hayes-Jordan et al. were able to show that disease-free survival also significantly increased in this patient group with a PCI<16 [20]. This correlation could not be shown in our report, which could be due to the different distribution of histologies in the two studies. In their cohort DSRCT accounted for the majority of cases at 42 %, followed by rhabdomyosarcoma at 14 % [20]. In contrast, adenocarcinomas predominate in our cohort, representing 72 % of the cases, reflecting a marked difference in tumor distribution between the two populations. Authors of the aforementioned comprehensive review observed favorable survival outcomes in patients with lower PCI scores, emphasizing the role of tumor burden in prognosis [8]. Again, attempting to extrapolate these prognostic factors to the subgroup of our CRC cases, the multivariable Cox regression on OS did not yield reasonable estimates due to small patient numbers.

In line with findings from other authors who similarly emphasized its pivotal role in improving long-term prognosis across various peritoneal malignancies, this data demonstrated that achieving complete cytoreduction is the most critical determinant of survival outcomes [20], 21]. In our cohort, patients with CCR-0 resections demonstrated significantly longer median OS compared to those with incomplete cytoreduction (CCR-2) (p=0.043). Similarly, Hayes-Jordan et al. observed a median OS of 31.4 months in patients with CCR-0/CCR-1, in contrast to just 7.1 months for those with CCR-2 [20]. Notably, patients with CCR-2 resections showed poorer outcomes, consistent with data from larger adult studies emphasizing the prognostic importance of complete cytoreduction [22], [23], [24], [25]. These comprehensive findings also indicate that patient selection prior surgery is of paramount importance. Although OS varies across different histological subtypes, achieving complete cytoreduction consistently results in improved survival outcomes, irrespective of tumor histology where tumor biology plays a crucial role [26].

The chemotherapeutic regimens used during HIPEC varied across the studies. In our cohort, cisplatin-based drugs were predominantly utilized. Both Hayes-Jordan et al. and Byrwa et al. reported using a wider variety of agents, including doxorubicin and mitomycin C, tailored to histopathologies such as DSRCT and mesothelioma [8], 14], 20], 27]. These differences highlight the variability in HIPEC protocols, driven by tumor-specific factors and institutional preferences, but emphasize the need for further standardization in pediatric populations. Nevertheless, in pediatric patients the most commonly used HIPEC drug is Cisplatin [8], 28] which demand greater attention due to its toxicity (renal, myelosuppression, hematologic toxicity, ototoxicity). There is sufficient data supporting the use of Sodium thiosulfate to reduce renal impairment and myelosuppression in both adult and pediatric individuals [27], 29], 30] and should be included in the protocols of children, similar to adults [30].

In our data, patients who received chemotherapy prior to surgery exhibited poorer OS compared to those who did not. However, the limited sample size in our cohort makes it challenging to draw definitive conclusions from this finding. To date, no data have been reported suggesting that neoadjuvant chemotherapy negatively impacts OS in pediatric patients undergoing CRS and HIPEC, as it is commonly integrated into treatment protocols without observed detrimental effects [31]. Furthermore, from our long term survivals three of seven have received previous chemotherapy prior to CRS and HIPEC.

CRS and HIPEC have proven to be a safe treatment approach for pediatric patients, as evidenced by the low rates of peri- and post-operative complications reported across multiple studies [13], 14], 29], 32]. In our cohort, the incidence of major complications Clavien–Dindo grade III or higher, was 24 %, which is slightly lower than the 28 % reported by Hayes-Jordan et al. in their study of pediatric patients undergoing CRS and HIPEC [20]. Furthermore, it should be emphasized in this context that while in adult patients the 30 days mortality rate ranges after CRS and HIPEC between 1.3 and 2.5 % [33], 34] in pediatric patients no perioperative mortality was reported likewise to our current findings, further emphasizing the safety of this multimodal treatment. Moreover, the complications reported such as pleural effusion or pneumonia were manageable and did not result in long-term morbidity [8].

The limitations of this study should be addressed. Due to its retrospective nature, the present study may contain biases. Due to the rarity of the presence of peritoneal metastases in pediatric patients, a long time period was chosen for the evaluation of patients. At the same time, there have been differences in oncology and HIPEC treatment protocols between centers over time. Furthermore, the heterogeneity of tumor histologies and therapies limits the internal consistency and interpretability of the study, including the conclusion about the value of neoadjuvant chemotherapy. Furthermore, the subgroup analyses by tumor type, age group and treatment variables are limited by the small number of cases and should be interpreted with caution. In the future, larger multicenter prospective studies are required to obtain more information from this patient population to gather best treatment strategies.

## Conclusions

These findings collectively highlight that, when performed by experienced multidisciplinary teams CRS and HIPEC represent a feasible and safe treatment modality for pediatric patients with peritoneal dissemination offering a favorable risk-benefit ratio.

Despite promising outcomes, the current body of literature is limited by small sample sizes and the retrospective nature of most analyses. Future multicenter, prospective studies are necessary to validate these findings and develop pediatric-specific CRS and HIPEC protocols, addressing histology-specific regimens, survivorship, and long-term outcomes.
